# Binge eating, depressive symptoms and suicidal ideation in obese candidates for bariatric surgery

**DOI:** 10.1007/s40519-023-01533-8

**Published:** 2023-02-17

**Authors:** Priscilla Vasconcelos Aguiar, Weslley Álex da Silva Dionisio, Ester Alana da Costa Souza, Davi Vantini, Raphael Campanholi, Tiago Coimbra Costa Pinto, Rosana Christine Cavalcanti Ximenes

**Affiliations:** 1 Postgraduate Program in Neuropsychiatry and Behavioral Sciences, Universidade Federal de Pernambuco, Recife, PE Brazil; 2 Centro Acadêmico de Vitória, Universidade Federal de Pernambuco, Vitória de Santo Antão, PE Brazil; 3 Clinical Analysis Laboratory, Faculdade de Medicina Do ABC, Centro Universitário FMABC, Santo André, SP Brazil

**Keywords:** Obesity, Binge eating disorder, Food addiction, Suicidal ideation, Depression

## Abstract

**Purpose:**

The aim of the present study was to determine possible associations between binge eating, depressive symptoms and suicidal ideation in obese candidates for bariatric surgery.

**Methods:**

A cross-sectional study was conducted with 254 obese patients recruited from the general surgery service for bariatric procedures at the hospital affiliated with the Federal University of Pernambuco, Brazil. Evaluations were performed using the Binge Eating Scale (BES), Beck Depression Inventory, Beck Scale for Suicidal Ideation (BSSI) and a questionnaire addressing sociodemographic characteristics.

**Results:**

Most patients were women (82%), 48% had a moderate binge eating disorder, 42% a severe binge eating disorder, 32% had symptoms suggestive of mild, moderate or severe depression and 6% had suicidal ideation. Severe binge eating was positively associated with depressive symptoms (*p* < 0.001) and suicidal ideation (*p* < 0.05). Cases of severe binge eating were more frequent in young adults, but not necessarily associated with symptoms of depression or suicidal ideation in this portion of the sample.

**Conclusions:**

The present findings underscore the need for psychological and psychiatric follow-up of obese candidates for bariatric surgery using appropriate assessment scales to guide therapeutic approaches.

**Level III:**

Evidence obtained from cross-sectional study.

**Supplementary Information:**

The online version contains supplementary material available at 10.1007/s40519-023-01533-8.

## Introduction

The global increase in the prevalence of obesity is directly related to economic and sociocultural changes in recent decades [1]. The predominance of activities and tasks requiring less physical effort, the increase in external stressors and the greater availability of foods with high palatability, low nutritional value and strong visual appeal are some of the factors responsible for the increase in obesity in the world population, the prevalence of which has nearly tripled since 1975 [2–4].

Individuals with obesity are not only exposed to obesity-associated diseases and higher mortality, but are also vulnerable to social prejudice and a wide array of psychological problems [3, 5]. The development of eating disorders, especially binge eating, is a common problem [6]. Binge eating can occur in people with healthy weight but is more common in overweight or obese individuals. Most binge eaters have a long history of dieting attempts and feel hopeless about their difficulty in controlling food intake [6, 7].

Binge eating disorder (BED) is one of the most common eating disorders in the obese population [8, 9]. BED is characterized by high food intake in a short period of time, a lack of control over food and consequent psychological suffering [10, 11]. Obese individuals with BED generally have more medical and psychiatric complications compared to non-compulsive obese individuals [11, 12].

Studies suggest that eating disorders may be more frequent in obese candidates for bariatric surgery as well as after this type of surgery [13, 14]. Binge eating prior to surgical intervention is associated with a worse prognosis, with a lower weight loss or the development of other disorders [5, 15, 16].

Among mental disorders, depressive symptoms classified are as one of the leading causes of disability in the world. Such symptoms are characterized by persistent sadness, feelings of guilt or low self-esteem, loss of interest or pleasure, altered sleep and appetite, lack of concentration and tiredness. Depressive symptoms can cause psychological suffering and dysfunction in all social spheres and can lead to extreme actions, such as suicide [10, 17].

Suicide is the fourth leading cause of death among individuals 15–29 years of age throughout the world. Suicidal behavior is defined as a set of thoughts and behaviors ranging from ideation to suicide itself [18]. Although cases of suicide have diminished on the global scale, suicide rates in the Americas increased by 17% between 2000 and 2019 [19]. Thus, whether or not associated with eating disorders, mental disorders affect the obese population, the most evident of which in this risk group are depressive symptoms and suicidal ideation [20, 21].

Although the literature has established prevalence rates and associations between binge eating disorder and both depressive symptoms and suicide risk, there is a gap with regards to these data among obese candidates for bariatric surgery. Few recent studies have been conducted on this issue. This gap is even more evident when considering Brazilian studies. Therefore, the aim of the present study was to evaluate associations between obesity, binge eating disorder, depressive symptoms and suicidal ideation in Brazilian candidates for bariatric surgery.

## Methods

### Study design and sample

An observational, analytical cross-sectional study was conducted at the hospital affiliated with the Federal University of Pernambuco, Brazil. The study sample consisted of 254 obese patients recruited from the general surgery department for bariatric surgery.

The inclusion criteria were male and female adults > 18 years of age) classified with obesity (body mass index (BMI) > 30 kg/m^2^) on the waiting list for bariatric surgery. Patients under 18 years of age and those with physical or cognitive impairments that made it impossible to answer the questionnaires were excluded. This study received approval from the Human Research Ethics Committee of the university (certificate number: 03925718.7.0000.5208). Data collection only began after approval from the ethics committee, authorization through a letter of consent, term of commitment and confidentiality and the signing of a statement of informed consent by the participants.

### Procedures, assessment tools and data collection

The main researcher conducted an active search for the names and records of patients on the waiting list for bariatric surgery, resulting in 254 candidates eligible to participate in the research. The patients were then contacted by telephone and those who agreed to participate in the study were scheduled for evaluation. All 254 patients agreed to participate and were included in the sample. The researchers underwent prior training conducted by a psychologist and the questionnaires were administered in the presence of a psychologist affiliated with the hospital. All questionnaires were validated and self-explanatory, requiring no intervention from the researcher. The recruitment and data collection phase was carried out in the pre-pandemic period in the second half of 2019, lasting five months.

A questionnaire based on the Brazilian Socioeconomic Classification Criteria [22] was used for the characterization of the sample. Data were collected on sociodemographic characteristics, such as sex, age, marital status, number of children, education level, family income, access to health care, region of origin, and recent psychological treatment.

The Binge Eating Scale (BES) is a self-administered questionnaire considered to be a valid tool for tracking BED [23] and has been translated and validated for the Brazilian population [23, 24]. The questionnaire has 16 items and 62 statements. For each item, the respondent selects the statement that best represents her or his situation. Each item is scored from 0 to 3 points and the total is the sum of the item scores. A score ≤ 17 points is considered indicative of an absence of BED, scores from 18 to 26 points are considered indicative of moderate BED and scores ≥ 27 points are considered indicative of severe BED [25].

The Beck Depression Inventory (BDI) is a self-report scale used to assess the intensity of depressive symptoms, which has been translated and validated for Brazilian adults [26]. This inventory consists of 21 items, with reliability established in six psychiatric samples [27]. Each item has four scored response options ranging from 0 to 3, from which the respondent chooses the most applicable to him/herself to describe how he/she has been feeling in the previous two weeks [28]. These items refer to increasing levels of depression severity. The total is calculated from the sum of the item scores, with a maximum of 63 points. The final score is classified into minimum, mild, moderate and severe levels, indicating the intensity of depression [29].

The Beck Scale for Suicidal Ideation (BSSI) was translated and adapted to Portuguese by Cunha [28]. The BSSI is used to detect suicidal ideation and measure the degree of motivation and planning of suicidal behavior. The scale comprises 21 items with statements scored from 0 to 2 points. The first 19 items address suicidal ideation, active suicide attempts, the desire to live, desire to die, reasons for living or dying, passive suicide attempts, duration of suicidal thoughts, frequency of suicidal ideation, control over suicidal ideation, obstacles to suicide attempts, attitude toward suicidal ideation, reasons for suicide attempts, specificity of plans, availability and opportunity to carry out the suicidal plan and ability to carry out the suicide attempt. Items 20 and 21 address the previous number of suicide attempts [30].

### Statistical analysis of data

Descriptive statistics was performed. Categorical variables were expressed as absolute and relative (%) frequencies. Age (only continuous variable were analyzed) was expressed as mean, standard deviation and median. The analytical statistics consisted of two phases: 1. Initially, Pearson's chi-square test or Fisher's exact test were used to assess possible associations between two categorical variables; 2. Variables that showed a significant association in the first stage were reassessed based on the total scores of the sample. Therefore, the Mann–Whitney U test and the Kruskal–Wallis test were used to evaluate significant correlations between variables evaluated. Homogeneity of variance was analyzed using Bartlett’s test. The margin of error used in decision of statistical tests was 5% (*p* < 0.05). All analyses were performed with the aid of IBM SPSS.

## Results

A total of 254 individuals participated in the study. Women accounted for more than 80% of the sample. Age ranged from 18 to 77 years (mean: 40 ± 12 years; median: 39 years). Most participants were either married (39.4%) or single (35.8%) (Similar portions of both groups) and had children (71.7%). The largest portion of the participants had a family income between one to two times the monthly minimum wage (49.2%) and most participants had a complete high school education. Only a small portion of the sample had access to private health care (5.1%) or recent psychological treatment (18.1%). The onset of obesity occurred in childhood for the largest portion of the sample, following by adulthood (Supplementary table 1).

Nearly 90% of the sample had some degree of BED and 42.1% had severe BED. The vast majority did not exhibit suicidal ideation and 31.9% had some risk of depressive symptoms. Among the participants with depressive symptoms, most had symptoms indicative of mild to moderate depression (Table 1).

**Table 1:** Distribution of clinical variables: binge eating, suicidal ideation and depressive symptoms

Variable	*N*	%
Total	254	100
Binge eating (BES)		
No binge eating	26	10.2
Moderate binge eating	121	47.6
Severe binge eating	107	42.1
Suicidal ideation (BSS)		
Yes	16	6.3
No	238	93.7
Depression (BDI)		
No depression risk	173	68.1
Mild depression	30	11.8
Moderate depression	30	11.8
Severe depression	21	8.3

Significant positive associations were found between severe BED and the prevalence of suicidal ideation and depressive symptoms (*p* < 0.05). 6.15% of the patients with BED presented suicidal ideation and 32.9% were positive for depressive symptoms. The percentage of patients without suicidal ideation or without depression risk was higher among patients with moderate or no BED. All degrees of depressive symptoms (from mild to severe) were more frequent in patients with severe binge eating, whereas the frequency of no risk of depression was lowest in this group (Table 2).

**Table 2:** Associations between binge eating and suicidal ideation and depressive symptoms

Variable	Binge eating scale								
	No binge eating		Moderate		Severe		Total		*p* value
	*N*	%	*n*	%	*N*	%	*n*	%	
Total cases	26	10.2	121	47.6	107	42.1	254	100.0	
Suicidal ideation (BSS)									*p*^(1)^ = 0.012*
Yes	2	7.7	2	2.0	12	11.2	16	6.3	
No	24	92.3	119	98.0	95	88.8	238	93.7	
Depression (BDI)									*p*^(2)^ < 0.001*
No depression risk	20	76.9	98	81.0	55	51.4	173	68.1	
Mild depression	2	7.7	12	9.9	16	15.0	30	11.8	
Moderate depression	4	15.4	5	4.1	21	19.6	30	11.8	
Severe depression	–	–	6	5.0	15	14.0	21	8.3	

(*) *p* < 0.05

(1) Pearson’s chi-squared test

(2) Fisher's exact test

To assess the consistency of the associations obtained by Pearson's chi-squared test and Fisher's exact test, an analysis was carried out on the total BED scores (BES) by group, according to the BDI and BSS classification. Therefore, it was observed that the associations between BED and symptoms of depression and suicidal ideation remained significant (*p* < 0.05).

Different BED scores were identified according to the severity of depressive symptoms [*X*^2^ (3) = 30.455; *p* < 0.001]. The group without risk of symptoms of depression had lower BED scores (Median = 23.00), when compared to groups with mild (Median = 32.50), moderate (Median = 32.00) and severe symptoms of depression (Median = 29.00).

When comparing the BED scores between positive and negative groups for suicidal ideation, it was possible to verify a statistically significant difference (*U* = 1344.500; *p* < 0.05; Mann–Whitney *U* test). Individuals who presented suicidal ideation (Median = 30.00; IR = 10) also had higher BED scores compared to the negative group for suicidal ideation (Median = 24.00; IR = 10) (Table 3).

**Table 3:** BED scores by group according to levels of depressive symptoms and presence of suicidal ideation

	Bing eating disorder						
	Average	SD	Median	IR	Minimum	Maximum	*p* value
Depression							*p*^(1)^ < 0.001*
No depression risk	24.13	6.06	23.00	9	9	45	
Mild depression	30.53	9.81	32.50	16	16	50	
Moderate depression	31.80	11.20	31.80	16	5	53	
Severe depression	30.19	7.43	29.00	11	18	45	
Suicidal ideation							*p*^(2)^ = 0.049*
Yes	29.31	9.75	30.00	10	5	44	
No	26.09	7.91	24.00	10	9	53	

*IR* interquartile range, *SD* standard deviation

(*) *p* < 0.05

(1) Kruskal-wallys’s test

(2) Test *U* of Mann–Whitney

When evaluated according to sex, symptoms of BED, depression and suicidal ideation did not show a significant association (*p* > 0.05). When analyzing clinical and sociographic variables, significant associations were found between age and degree of BED, between recent psychological treatment and suicidal ideation and between not having children and depressive symptoms (*p* = 0.001, *p* = 0.013 and *p* = 0.048, respectively) (Supplementary tables).

## Discussion

According to current data, more than one billion people in the world are obese and the World Health Organization estimates that approximately 167 million people will be less healthy by the year 2025 due to being overweight or obese. Obesity is one of the main adverse health conditions capable of compromising physical and mental health and is directly related to psychiatric and eating disorders [1, 8, 10]. Therefore, the present study evaluated associations between BED, depressive symptoms and suicidal ideation in obese candidates for bariatric surgery.

### Binge eating and obesity

In a survey conducted with 24,124 individuals 18 years of age or older in 14 different countries (2,942 Brazilians), the prevalence of BED was 1.9% throughout life and 0.8% in the previous 12 months. When considering only those with obesity, these figures were respectively 36.2% and 41.7% [31]. More recent epidemiological studies involving obese candidates for bariatric surgery reveal updated percentages for this population.

Evaluating 92 patients, all obese patients who were candidates for bariatric surgery, (81.5% women) with a mean BMI of 41.3 ± 0.6 km/m^2^ and mean age of 32 ± 1.5 years, Da Silva et al. [32] found that the prevalence of BED was 32.6% ± 5%. In a Brazilian study involving 281 obese candidates for bariatric surgery in the state of São Paulo, 32% had symptoms suggestive of BED [33]. In a study involving 121 French obese candidates for bariatric surgery individuals between 19 and 59 years of age (79.3% women; mean age: 40.82 ± 9.26 years; mean BMI: 44.92 ± 7.55 kg/m^2^) conducted between November 2017 and October 2018, the prevalence of BED (evaluated using the BES) was 22.31% [34]. In the present investigation, the prevalence of BED among obese candidates for bariatric surgery was higher than the rates reported in the literature, as 89.7% of the sample had symptoms of moderate to severe BED. Moreover, approximately half of this group (42.7%) had symptoms of severe BED.

In addition to offering more current data, as data collection in the present study was carried out in a more recent period than that of other studies and this study was conducted in the Northeast region of Brazil. The population in this region is characterized by the consumption of palatable high-calorie foods as well as the intake of a large amount of food per meal. These characteristic sociocultural factors of the region may have contributed to a higher rate of BED in the sample [35, 36].

The association between obesity and BED has been known and studied for a long time. This association is explained by mechanisms involved in the onset and maintenance of the two conditions, which have similar intrinsic and extrinsic associated factors [37]. A classic study conducted with a population-based sample of 2613 twins and published around 20 years ago indicated the both obesity and BED share some genetic and environmental factors. According to the authors, the development of both conditions may be influenced by hereditary factors [38]. In the literature, adverse events in life, especially traumatic experiences, are among the main factors associated with the development of obesity and BED [39], indicating that both conditions have a multifactorial etiology.

The risk of BED tends to be directly proportional to the excess weight of the individual [12]. Evidence shows that the maintenance of body weight as and compulsive food intake are associated with the functioning of brain pathways of adequate inhibitory control and impulsivity [40, 41].

Obese individuals generally have decreased activity in brain regions associated with inhibitory control (prefrontal cortex, parietal cortex, insula, cuneus and supplementary motor area) [40, 41] as well as altered activity in regions associated with satiety, pleasure, appetite and the mediation of responses to food stimuli (striatum) [37]. This can lead to an increase in impulsivity toward food intake [37, 42–44]. Such impulsivity is generally reported as one of the main factors that make obese individuals susceptible to the development of BED [44].

Therefore, it is essential to evaluate the symptoms of BED in obese patients, especially in those who will undergo bariatric surgery. Although evidence already points to psychological benefits acquired with bariatric surgery (increased self-esteem, improved social relationships, decreased anxiety and depression), BED symptoms may persist after the procedure in these patients. In addition, bariatric surgery itself causes several endocrines and gastrointestinal changes that can be aggravated if binge eating behavior persists [45].

### Associations between binge eating, depressive symptoms and suicidal ideation in obese individuals

Studies have reported associations between symptoms of BED and psychiatric comorbidities, including depressive symptoms. In a representative study conducted in the United States, 94% of individuals with BED reported mental health symptoms throughout life and 70% had mood disorders [46]. Moreover, the increase in BMI seems to be an aggravating factor of these symptoms. There are also reports of an association between preoperative depressive symptoms and the diagnosis as well as the severity of self-reported binge eating among candidates for bariatric surgery.

High rates of general depressive symptoms (58%) [33] and major depression disorder (32.6%) [32] have been reported in obese candidates for bariatric surgery. Besides the association between the diseases, the severity of depressive symptoms has also been associated with binge eating behavior [34].

A study conducted in 2020 with 345 volunteers involving the administration of the Beck Depression Inventory found that those who reported atypical depressive symptoms were 10.1-fold more likely to report clinically significant binge eating severity. The study also reported that candidates for bariatric surgery who reported atypical depressive symptoms were at greater risk of a diagnosis of BED and a greater severity of the disorder [47].

In agreement with most studies published, the present investigation identified that 32.9% of the individuals with BED had some degree of depressive symptoms (*p* < 0.05) and the prevalence of moderate and severe symptoms was 11.4% and 9.21%, respectively. Moreover, significant associations were found between severe BED and the prevalence of suicidal and ideation and depressive symptoms in candidates for bariatric surgery, which is in agreement with results described in the study conducted by Smith et al. We also found that moderate to severe depressive symptoms were more frequent in patients with severe BED.

These findings may be explained by the increase in impulsivity generated by morphofunctional changes in the brain associated with a higher frequency of depressive symptoms and a greater risk of suicide in the obese population with BED. Food dependence, which is directly associated with BED, seems to be related to a high frequency of “emotional eating”, depressive symptoms and current suicidal tendency [48].

Besides impulsivity with regards to food, individuals with BED tend to have less emotional control in the occurrence of adverse events. Thus, when such dysregulation precedes episodes of excessive food intake, which is characteristic of BED, individuals tend to have feelings of guilt and embarrassment associated with the lack of control, which may favor a vicious cycle of depressive symptoms and uncontrolled food intake [12, 49].

Another aspect to consider is that individuals with depressive symptoms dedicate considerable time to sedentary activities and are more prone to emotional eating, which often leads to excess energy intake, favoring obesity [50]. Depressive symptoms may be present for long periods of time or may be recurrent, substantially compromising the capacity to be functional at work or school and to deal with daily living. In its most extreme state, such symptoms can lead to suicide [1].

The literature reports an associated with between BMI and suicidal tendency. Moreover, BED has the potential to strengthen this association. This may be explained by the association between BED and an increase in BMI with the reduction in inhibitory control and an increase in impulsivity generated by changes in the activity of regions of the brain, such as frontal regions and frontostriatal pathways [37, 40, 44], which are also altered in individuals with suicidal ideation or behavior [51–53].

A study involving the evaluation of 14,497 individuals recruited for the Collaborative Psychiatric Epidemiologic Surveys (CPES, 2001–2003) found that 37.5% of those with BED reported having thoughts about suicide, 20.5% had attempted suicide and 8.9% attempted suicide in the year prior to the study. Similar rates were found for individuals who did not meet all diagnostic criteria for BED but had a history of binge eating (34.2, 18.6 and 10.1%, respectively). Thus, even when not yet denoting the disorder, recurrent binge eating behavior constitutes a high risk of suicide [54]. The prevalence of suicidal ideation was lower in the present study (6.14%) compared to figures reported in the literature, but 75% of the individuals who reported suicidal ideation had symptoms of severe BED.

From this perspective, it is possible to establish that BED, in addition to being closely linked to obesity, can have severe psychiatric implications. Therefore, although bariatric surgery is considered by many to be the most effective obesity treatment, there is already evidence that proves a high prevalence of depressive symptoms and suicidal ideation in patients after the procedure. According to the literature, the persistence of both conditions after surgery may be associated with weight regain, eating disorders, and quality of life in these individuals [55, 56]. It is suggested, from this, that the most indicated in this case would be to conduct a complementary evaluation, and possible intervention, about the presence of depressive symptoms and suicidal ideation in obese patients with BED before and after bariatric surgery.

### Associations between clinical and sociodemographic variables

Sociodemographic characteristics of the present sample were associated with clinical variables. Most individuals reported the onset of obesity in childhood or adulthood. The prevalence of excess weight in Brazil is 7.2% in children less than five years of age, 34.8% among children five to nine years of age and 25.5% among adolescents 12–17 years of age. The projection of the impact of childhood obesity shows that children with obesity at two years of age have a 75% chance of being obese at 35 years and adolescents with obesity at 19 years of age have an 89% of remaining obese at 35 years, which is not necessarily related to binge eating in childhood [57].

Another finding of the present study was that individuals with children had a lower risk of depressive symptoms, whereas those without children were more prevalent in the group with moderate symptoms. The age group of 46 years or older was more frequent among individuals with without BED or with moderate BED compared to other two age groups, whereas the percentage of participants with severe BED was higher in the group of young adults. These findings are in agreement with data reported in some previous studies. In comparison to older individuals, young adults are exposed to a greater quantity of risk factors related to their bodies and body image, which increases the risk of the development of eating disorders and psychiatric disorders, including suicide, in this population [58].

Suicidal ideation was more frequent among participants in recent psychological treatment compared to those who were not in psychotherapy. In addition to this, 81.9% of participants reported not having undergone recent psychological treatment. According to the Brazilian Ministry of Health, psychological support is one of the essential criteria for bariatric surgery [59]. However, some health services only offer a pre-clinical evaluation to verify the patient's eligibility for a possible surgery, leaving the patient to search for a specialized psychological service independently.

The association between socioeconomic status and obesity is widely documented in the literature. An important systematic review with meta-analysis evaluated 14 studies and found a consistent association between a lower socioeconomic status and obesity in women [60]. Likewise, 72.8% of the sample in the present study was composed of individuals with a low income (up to two times the monthly minimum wage).

Moreover, the difficulty in gaining access to a healthcare system of quality may exert an influence on the maintenance of poor dietary habits and psychiatric symptoms in this population. The public healthcare system in Brazil is saturated due to overpopulation and the wait for a medical appointment is quite long, limiting rapid intervention and an efficient prevention policy directed at obesity and associated risks. It is therefore necessary to outline strategies capable of favoring access to adequate treatment of quality for obese individuals with BED and determine what therapeutic tools could be effective at improving treatment for these individuals.

#### Strengths and limitations

The main limitation of the present study is the fact that nearly 80% of the sample was comprised of women, with no significant data presented for the male sex. However, the literature demonstrates that the prevalence of obesity and BED is higher among women compared to men [50, 54]. Moreover, the rates demonstrated and the greater number of women in studies may be influenced by the fact that women seek healthcare services more often than men.

Individuals who seek treatment for obesity may attempt to mask their symptoms due to fear of not receiving the desired bariatric surgery. Thus, they may not be completely sincere when answering questionnaires to hide their emotional state, which can lead to an underestimation of the actual number of cases of binge eating, depression and suicidal ideation, constituting a source of potential bias in this study. However, the participants were assured of the confidentiality of their data during the administration of the questionnaires to minimize this risk.

Another limitation regards the use of screening tools for the determination of the variables of interest. However, all questionnaires used in this study had been translated and validated for the Brazilian population and have adequate psychometric properties for the public evaluated. Lastly, the sample was relatively small sample for a cross-sectional study. However, investigations with obese candidates for bariatric surgery tend to have small samples due to the fact that such studies are generally conducted at specialized hospitals or treatment centers that work with this population. Indeed, the sample in the present study is similar to or larger than that of most recent studies published with this population.

#### What is already known on this subject?

Obesity is currently one of the chronic diseases that most affect the world population precisely due to its association with poor habits and behaviors. The increase body weight is accompanied by changes in regions of the brain that predispose individuals to the development of binge eating disorder. Moreover, this disorder itself may intensify such changes, exposing individuals to depressive symptoms and the risk of suicide.

#### What this study adds?

Data on the suicide rate in the population with binge eating disorder are scarce [46]. The present study offers important findings for the understanding of psychiatric symptoms, including the risk of suicide, in obese individuals with binge eating disorder. This study is essential when we consider the low quantity of information on the variables analyzed in the Brazilian context, considering the high frequency of obesity and the number of individuals without adequate care in the country. Even though it is considered one of the most effective treatments for obesity, the clinical management of bariatric surgery patients generally focuses on weight loss and improvement of obesity-related conditions. However, a satisfactory result of the method in question is also dependent on the improvement of mental health status [55, 56]. In addition, it is suggested that the patient undergo a mental health assessment before and after the patient undergoes surgery.

## Conclusion

Most of the individuals analyzed in the present study had moderate or severe binge eating disorder. Severe cases were more frequent among younger adults, but not necessarily related to depressive symptoms or suicide ideation in this portion of the sample. Mild to moderate depressive symptoms were associated with obesity. However, severe binge eating disorder and all levels of depressive symptoms were associated with suicidal ideation, underscoring the need for intensive psychiatric and psychological support to avoid the occurrence of suicide.

## Supplementary Information

Below is the link to the electronic supplementary material.

Supplementary file1 (DOCX 56 KB)

## Data Availability

All data and materials support the published statements and meet field standards.
