# Supplementary material for: Binge eating, depressive symptoms and suicidal ideation in obese candidates for bariatric surgery
Source: Eat Weight Disord. 2023 Feb 17;28(1):12. doi: 10.1007/s40519-023-01533-8 (PMC9938051; doi:10.1007/s40519-023-01533-8)
Supplement: Supplementary file 1 — Supplementary file1 (DOCX 56 KB) [file 40519_2023_1533_MOESM1_ESM.docx]

**SUPPLEMENTARY FILES**

**Supplementary Table 1**. Distribution of sociodemographic variables in sample.

|  |  |  |
| --- | --- | --- |
| **Variable** | **N** | **%** |
|  |  |  |
|  |  |  |
| **TOTAL** | **254** | **100** |
|  |  |  |
| **Sex** |  |  |
| Male | 45 | 17.7 |
| Female | 209 | 82.3 |
|  |  |  |
| **Age (years)** |  |  |
| 18 to 35 | 93 | 36.6 |
| 36 to 45 | 71 | 28.0 |
| ≥ 46 | 82 | 32.3 |
| Not reported | 8 | 3.1 |
|  |  |  |
| **Marital status** |  |  |
| Single | 91 | 35.8 |
| Married | 100 | 39.4 |
| Stable union | 9 | 3.5 |
| Divorced | 10 | 3.9 |
| Not reported | 44 | 17.3 |
|  |  |  |
| **Access to health care** |  |  |
| Private service | 13 | 5.1 |
| Public service | 197 | 77.6 |
| Not reported | 44 | 17.3 |
|  |  |  |
| **Region of origin** |  |  |
| Metropolitan Recife | 114 | 44.9 |
| Semiarid region of state (PE) | 85 | 33.5 |
| Other regions of PE | 11 | 4.3 |
| Not informed | 44 | 17.3 |
|  |  |  |
| **Income** |  |  |
| < monthly minimum wage | 60 | 23.6 |
| ≥1 to <2 x monthly minimum wage | 125 | 49.2 |
| ≥2 to <5 x monthly minimum wage | 22 | 8.7 |
| ≥5 to ≤10 monthly minimum wage | 3 | 1.2 |
| Not reported | 44 | 17.3 |
|  |  |  |
| **Schooling** |  |  |
| Incomplete primary school | 28 | 11 |
| Elementary school | 64 | 25.2 |
| High school | 133 | 52.4 |
| University/college | 29 | 11.4 |
|  |  |  |
| **Obesity onset** |  |  |
| Childhood | 83 | 32.7 |
| Adolescence | 49 | 19.3 |
| Adulthood | 60 | 23.6 |
| Childhood and Adulthood | 1 | 0.4 |
| Not reported | 61 | 24.0 |
|  |  |  |
| **Children** |  |  |
| Yes | 182 | 71.7 |
| No | 72 | 28.3 |
|  |  |  |
| **Recent psychological treatment** |  |  |
| Yes | 46 | 18.1 |
| No | 208 | 81.9 |
|  |  |  |

PE: state of Pernambuco, Brazil.

**Supplementary Table 2.** Binge eating scale results according to sociodemographic characteristics

|  | | **Binge Eating Scale** | | | | |  | | | | |  | | | |
| --- | --- | --- | --- | --- | --- | --- | --- | --- | --- | --- | --- | --- | --- | --- | --- |
| **Variable** | **No binge eating** | | | **Moderate** | | | **Severe** | | | **Total** | | | | ***p*-value** | |
|  | **N** | | **%** | | **N** | **%** | | **N** | **%** | | **N** | | **%** | |  |
|  |  | |  | |  |  | |  |  | |  | |  | |  |
| **Sex** |  | |  | |  |  | |  |  | |  | |  | | p^(1)^ = 0.943 |
| Male | 5 | | 11.1 | | 22 | 48.9 | | 18 | 40.0 | | 45 | | 100.0 | |  |
| Female | 21 | | 10.0 | | 99 | 47.4 | | 89 | 42.6 | | 209 | | 100.0 | |  |
| **Total** | **26** | | **10.2** | | **121** | **47.6** | | **107** | **42.1** | | **254** | | **100.0** | |  |
|  |  | |  | |  |  | |  |  | |  | |  | |  |
| **Age (Years)** |  | |  | |  |  | |  |  | |  | |  | | p^(1)^ = 0.001* |
| 18 to 35 | 5 | | 5.4 | | 37 | 39.8 | | 51 | 54.8 | | 93 | | 100.0 | |  |
| 36 to 45 | 5 | | 7.0 | | 36 | 50.7 | | 30 | 42.3 | | 71 | | 100.0 | |  |
| ≥46 | 14 | | 17.1 | | 47 | 57.3 | | 21 | 25.6 | | 82 | | 100.0 | |  |
| **Total** | **24** | | **9.8** | | **120** | **48.8** | | **102** | **41.5** | | **246** | | **100.0** | |  |
|  |  | |  | |  |  | |  |  | |  | |  | |  |
| **Marital State** |  | |  | |  |  | |  |  | |  | |  | | p^(1)^ = 0.156 |
| Single/ Divorced | 10 | | 9.9 | | 44 | 43.6 | | 47 | 46.5 | | 101 | | 100.0 | |  |
| Married/Stable Union | 16 | | 14.7 | | 56 | 51.4 | | 37 | 33.9 | | 109 | | 100.0 | |  |
| **Total** | **26** | | **12.4** | | **100** | **47.6** | | **84** | **40.0** | | **210** | | **100.0** | |  |
|  |  | |  | |  |  | |  |  | |  | |  | |  |
| **Access to Health Care** |  | |  | |  |  | |  |  | |  | |  | | p^(1)^ = 0.561 |
| Private service | 1 | | 7.7 | | 5 | 38.5 | | 7 | 53.8 | | 13 | | 100.0 | |  |
| Public service | 25 | | 12.7 | | 95 | 48.2 | | 77 | 39.1 | | 197 | | 100.0 | |  |
| **Total** | **26** | | **12.4** | | **100** | **47.6** | | **84** | **40.0** | | **210** | | **100.0** | |  |
|  |  | |  | |  |  | |  |  | |  | |  | |  |
| **Region of Origin** |  | |  | |  |  | |  |  | |  | |  | | p^(2)^ = 0.544 |
| Metropolitan Recife | 14 | | 12.3 | | 58 | 50.9 | | 42 | 36.8 | | 114 | | 100.0 | |  |
| Semi-arid region of state | 11 | | 12.9 | | 35 | 41.2 | | 39 | 45.9 | | 85 | | 100.0 | |  |
| Other regions of state | 1 | | 9.1 | | 7 | 63.6 | | 3 | 27.3 | | 11 | | 100.0 | |  |
| **Total** | **26** | | **12.4** | | **100** | **47.6** | | **84** | **40.0** | | **210** | | **100.0** | |  |
|  |  | |  | |  |  | |  |  | |  | |  | |  |
| **Income** |  | |  | |  |  | |  |  | |  | |  | | p^(1)^ = 0.680 |
| < monthly min. wage | 8 | | 13.3 | | 27 | 45.0 | | 25 | 41.7 | | 60 | | 100.0 | |  |
| ≥1 to <2 x monthly min. wage | 16 | | 12.8 | | 63 | 50.4 | | 46 | 36.8 | | 125 | | 100.0 | |  |
| ≥2 to <10 x monthly min. wage | 2 | | 8.0 | | 10 | 40.0 | | 13 | 52.0 | | 25 | | 100.0 | |  |
| **Total** | **26** | | **12.4** | | **100** | **47.6** | | **84** | **40.0** | | **210** | | **100.0** | |  |
|  |  | |  | |  |  | |  |  | |  | |  | |  |
| **Schooling** |  | |  | |  |  | |  |  | |  | |  | | p^(1)^ = 0.550 |
| Incomplete primary school | 3 | | 10.7 | | 16 | 57.1 | | 9 | 32.1 | | 28 | | 100.0 | |  |
| Primary school | 10 | | 15.6 | | 26 | 40.6 | | 28 | 43.8 | | 64 | | 100.0 | |  |
| High school | 11 | | 8.3 | | 66 | 49.6 | | 56 | 42.1 | | 133 | | 100.0 | |  |
| University/college | 2 | | 6.9 | | 13 | 44.8 | | 14 | 48.3 | | 29 | | 100.0 | |  |
| **Total** | **26** | | **10.2** | | **121** | **47.6** | | **107** | **42.1** | | **254** | | **100.0** | |  |
|  |  | |  | |  |  | |  |  | |  | |  | |  |
| **Obesity onset** |  | |  | |  |  | |  |  | |  | |  | | p^(2)^ = 0.519 |
| Childhood | 10 | | 12.0 | | 43 | 51.8 | | 30 | 36.1 | | 83 | | 100.0 | |  |
| Adolescence | 6 | | 12.2 | | 24 | 49.0 | | 19 | 38.8 | | 49 | | 100.0 | |  |
| Adulthood | 6 | | 10.0 | | 23 | 38.3 | | 31 | 51.7 | | 60 | | 100.0 | |  |
| Childhood and Adulthood | - | | - | | 1 | 100.0 | | - | - | | 1 | | 100.0 | |  |
| **Total** | **22** | | **11.4** | | **91** | **47.2** | | **80** | **41.5** | | **193** | | **100.0** | |  |
|  |  | |  | |  |  | |  |  | |  | |  | |  |
| **Children** |  | |  | |  |  | |  |  | |  | |  | | p^(1)^ = 0.975 |
| Yes | 19 | | 10.4 | | 87 | 47.8 | | 76 | 41.8 | | 182 | | 100.0 | |  |
| No | 7 | | 9.7 | | 34 | 47.2 | | 31 | 43.1 | | 72 | | 100.0 | |  |
| **Total** | **26** | | **10.2** | | **121** | **47.6** | | **107** | **42.1** | | **254** | | **100.0** | |  |
|  |  | |  | |  |  | |  |  | |  | |  | |  |
| **Recent psychological treatment** |  | |  | |  |  | |  |  | |  | |  | | p^(1)^ = 0.311 |
| Yes | 2 | | 4.3 | | 22 | 47.8 | | 22 | 47.8 | | 46 | | 100.0 | |  |
| No | 24 | | 11.5 | | 99 | 47.6 | | 85 | 40.9 | | 208 | | 100.0 | |  |
| **Total** | **26** | | **10.2** | | **121** | **47.6** | | **107** | **42.1** | | **254** | | **100.0** | |  |
|  |  | |  | |  |  | |  |  | |  | |  | |  |

(*) P<0.05

(**) not determined due to null frequency

(1) Pearson’s chi-squared test

(2) Fisher's exact test

**Supplementary Table 3.** Results of Beck Scale for Suicidal Ideation (BSS) according to sociodemographic characteristics

|  | **Beck Scale for Suicidal Ideation** | | | | | | |  |
| --- | --- | --- | --- | --- | --- | --- | --- | --- |
| **Variable** | **Yes** | | **No** | | | **Total** | | **p-value** |
|  | **N** | **%** | **N** | **%** | **n** | | **%** |  |
|  |  |  |  |  |  | |  |  |
| **Sex** |  |  |  |  |  | |  | p^(1)^ = 1.000 |
| Male | 3 | 6.7 | 42 | 93.3 | 45 | | 100.0 |  |
| Female | 13 | 6.2 | 196 | 93.8 | 209 | | 100.0 |  |
| **Total** | **16** | **6.3** | **238** | **93.7** | **254** | | **100.0** |  |
|  |  |  |  |  |  | |  |  |
| **Age (Years)** |  |  |  |  |  | |  | p^(2)^ = 0.572 |
| 18 to 35 | 8 | 8.6 | 85 | 91.4 | 93 | | 100.0 |  |
| 36 to 45 | 4 | 5.6 | 67 | 94.4 | 71 | | 100.0 |  |
| ≥46 | 4 | 4.9 | 78 | 95.1 | 82 | | 100.0 |  |
| **Total** | **16** | **6.5** | **230** | **93.5** | **246** | | **100.0** |  |
|  |  |  |  |  |  | |  |  |
| **Marital State** |  |  |  |  |  | |  | p^(2)^ = 0.156 |
| Single/ Divorced | 3 | 3.0 | 98 | 97.0 | 101 | | 100.0 |  |
| Married/Stable Union | 8 | 7.3 | 101 | 92.7 | 109 | | 100.0 |  |
| **Total** | **11** | **5.2** | **199** | **94.8** | **210** | | **100.0** |  |
|  |  |  |  |  |  | |  |  |
| **Access to Health Care** |  |  |  |  |  | |  | p^(1)^ = 0.514 |
| Private service | 1 | 7.7 | 12 | 92.3 | 13 | | 100.0 |  |
| Public service | 10 | 5.1 | 187 | 94.9 | 197 | | 100.0 |  |
| **Total** | **11** | **5.2** | **199** | **94.8** | **210** | | **100.0** |  |
|  |  |  |  |  |  | |  |  |
| **Region of Origin** |  |  |  |  |  | |  | p^(1)^ = 1.000 |
| Metropolitan Recife | 6 | 5.3 | 108 | 94.7 | 114 | | 100.0 |  |
| Semi-arid region of state | 5 | 5.9 | 80 | 94.1 | 85 | | 100.0 |  |
| Other regions of state | - | - | 11 | 100.0 | 11 | | 100.0 |  |
| **Total** | **11** | **5.2** | **199** | **94.8** | **210** | | **100.0** |  |
|  |  |  |  |  |  | |  |  |
| **Income** |  |  |  |  |  | |  | p^(1)^ = 0.542 |
| < monthly min. wage | 4 | 6.7 | 56 | 93.3 | 60 | | 100.0 |  |
| ≥1 to <2 x monthly min. wage | 7 | 5.6 | 118 | 94.4 | 125 | | 100.0 |  |
| ≥2 to <10 x monthly min. wage | - | - | 25 | 100.0 | 25 | | 100.0 |  |
| **Total** | **11** | **5.2** | **199** | **94.8** | **210** | | **100.0** |  |
|  |  |  |  |  |  | |  |  |
| **Schooling** |  |  |  |  |  | |  | p^(1)^ = 0.857 |
| Incomplete primary school | 2 | 7.1 | 26 | 92.9 | 28 | | 100.0 |  |
| Primary school | 5 | 7.8 | 59 | 92.2 | 64 | | 100.0 |  |
| High school | 8 | 6.0 | 125 | 94.0 | 133 | | 100.0 |  |
| University/college | 1 | 3.4 | 28 | 96.6 | 29 | | 100.0 |  |
| **Total** | **16** | **6.3** | **238** | **93.7** | **254** | | **100.0** |  |
|  |  |  |  |  |  | |  |  |
| **Obesity onset** |  |  |  |  |  | |  | p^(1)^ = 0.447 |
| Childhood | 3 | 3.6 | 80 | 96.4 | 83 | | 100.0 |  |
| Adolescence | 2 | 4.1 | 47 | 95.9 | 49 | | 100.0 |  |
| Adulthood | 5 | 8.3 | 55 | 91.7 | 60 | | 100.0 |  |
| Childhood and Adulthood | - | - | 1 | 100.0 | 1 | | 100.0 |  |
| **Total** | **10** | **5.2** | **183** | **94.8** | **193** | | **100.0** |  |
|  |  |  |  |  |  | |  |  |
| **Children** |  |  |  |  |  | |  | p^(1)^ = 0.779 |
| Yes | 11 | 6.0 | 171 | 94.0 | 182 | | 100.0 |  |
| No | 5 | 6.9 | 67 | 93.1 | 72 | | 100.0 |  |
| **Total** | **16** | **6.3** | **238** | **93.7** | **254** | | **100.0** |  |
|  |  |  |  |  |  | |  |  |
| **Recent psychological treatment** |  |  |  |  |  | |  | p^(1)^ = 0.013* |
| Yes | 7 | 15.2 | 39 | 84.8 | 46 | | 100.0 |  |
| No | 9 | 4.3 | 199 | 95.7 | 208 | | 100.0 |  |
| **Total** | **16** | **6.3** | **238** | **93.7** | **254** | | **100.0** |  |
|  |  |  |  |  |  | |  |  |

(*) P<0.05

(**) not determined due to null frequency

(1) Pearson’s chi-squared test

(2) Fisher's exact test

**Supplementary Table 4**. Results of Beck Depression Inventory (BDI) according to sociodemographic characteristics

|  | **Beck Depression Inventory** | | | | | | | |  |  |  |
| --- | --- | --- | --- | --- | --- | --- | --- | --- | --- | --- | --- |
| **Variable** | **No risk** | | **Mild** | | **Moderate** | | **Severe** | | **Total** | | ***p*-value** |
|  | **n** | **%** | **n** | **%** | **n** | **%** | **n** | **%** | **n** | **%** |  |
|  |  |  |  |  |  |  |  |  |  |  |  |
| **Sex** |  |  |  |  |  |  |  |  |  |  | p^(1)^ = 0.367 |
| Male | 32 | 71.1 | 5 | 11.1 | 7 | 15.6 | 1 | 2.2 | 45 | 100.0 |  |
| Female | 141 | 67.5 | 25 | 12.0 | 23 | 11.0 | 20 | 9.6 | 209 | 100.0 |  |
| **Total** | **173** | **68.1** | **30** | **11.8** | **30** | **11.8** | **21** | **8.3** | **254** | **100.0** |  |
|  |  |  |  |  |  |  |  |  |  |  |  |
| **Age (Years)** |  |  |  |  |  |  |  |  |  |  | p^(1)^ = 0.279 |
| 18 to 35 | 17 | 60.7 | 1 | 3.6 | 8 | 28.6 | 2 | 7.1 | 28 | 100.0 |  |
| 36 to 45 | 40 | 62.5 | 11 | 17.2 | 6 | 9.4 | 7 | 10.9 | 64 | 100.0 |  |
| ≥46 | 116 | 71.6 | 18 | 11.1 | 16 | 9.9 | 12 | 7.4 | 162 | 100.0 |  |
| **Total** | **173** | **68.1** | **30** | **11.8** | **30** | **11.8** | **21** | **8.3** | **254** | **100.0** |  |
|  |  |  |  |  |  |  |  |  |  |  |  |
| **Marital State** |  |  |  |  |  |  |  |  |  |  | p^(1)^ = 0.179 |
| Single/ Divorced | 67 | 66.3 | 16 | 15.8 | 11 | 10.9 | 7 | 6.9 | 101 | 100.0 |  |
| Married/Stable Union | 79 | 72.5 | 7 | 6.4 | 13 | 11.9 | 10 | 9.2 | 109 | 100.0 |  |
| **Total** | **146** | **69.5** | **23** | **11.0** | **24** | **11.4** | **17** | **8.1** | **210** | **100.0** |  |
|  |  |  |  |  |  |  |  |  |  |  |  |
| **Access to Health Care** |  |  |  |  |  |  |  |  |  |  | p^(2)^ = 0.588 |
| Private service | 8 | 61.5 | 1 | 7.7 | 2 | 15.4 | 2 | 15.4 | 13 | 100.0 |  |
| Public service | 138 | 70.1 | 22 | 11.2 | 22 | 11.2 | 15 | 7.6 | 197 | 100.0 |  |
| **Total** | **146** | **69.5** | **23** | **11.0** | **24** | **11.4** | **17** | **8.1** | **210** | **100.0** |  |
|  |  |  |  |  |  |  |  |  |  |  |  |
| **Region of Origin** |  |  |  |  |  |  |  |  |  |  | p^(2)^ = 0.238 |
| Metropolitan Recife | 82 | 71.9 | 9 | 7.9 | 16 | 14.0 | 7 | 6.1 | 114 | 100.0 |  |
| Semi-arid region of state | 54 | 63.5 | 13 | 15.3 | 8 | 9.4 | 10 | 11.8 | 85 | 100.0 |  |
| Other regions of state | 10 | 90.9 | 1 | 9.1 | 0 | 0.0 | 0 | 0.0 | 11 | 100.0 |  |
| **Total** | **146** | **69.5** | **23** | **11.0** | **24** | **11.4** | **17** | **8.1** | **210** | **100.0** |  |
|  |  |  |  |  |  |  |  |  |  |  |  |
| **Income** |  |  |  |  |  |  |  |  |  |  | p^(2)^ = 0.464 |
| < monthly min. wage | 40 | 66.7 | 4 | 6.7 | 9 | 15.0 | 7 | 11.7 | 60 | 100.0 |  |
| ≥1 to <2 x monthly min. wage | 85 | 68.0 | 17 | 13.6 | 14 | 11.2 | 9 | 7.2 | 125 | 100.0 |  |
| ≥2 to <10 x monthly min. wage | 21 | 84.0 | 2 | 8.0 | 1 | 4.0 | 1 | 4.0 | 25 | 100.0 |  |
| **Total** | **146** | **69.5** | **23** | **11.0** | **24** | **11.4** | **17** | **8.1** | **210** | **100.0** |  |
|  |  |  |  |  |  |  |  |  |  |  |  |
| **Schooling** |  |  |  |  |  |  |  |  |  |  | p^(2)^ = 0.115 |
| Incomplete primary school | 17 | 60.7 | 1 | 3.6 | 8 | 28.6 | 2 | 7.1 | 28 | 100.0 |  |
| Primary school | 40 | 62.5 | 11 | 17.2 | 6 | 9.4 | 7 | 10.9 | 64 | 100.0 |  |
| High school | 93 | 69.9 | 14 | 10.5 | 14 | 10.5 | 12 | 9.0 | 133 | 100.0 |  |
| University/college | 23 | 79.3 | 4 | 13.8 | 2 | 6.9 | 0 | 0.0 | 29 | 100.0 |  |
| **Total** | **173** | **68.1** | **30** | **11.8** | **30** | **11.8** | **21** | **8.3** | **254** | **100.0** |  |
|  |  |  |  |  |  |  |  |  |  |  |  |
| **Obesity onset** |  |  |  |  |  |  |  |  |  |  | p^(2)^ = 0.626 |
| Childhood | 59 | 71.1 | 9 | 10.8 | 10 | 12.0 | 5 | 6.0 | 83 | 100.0 |  |
| Adolescence | 33 | 67.3 | 5 | 10.2 | 6 | 12.2 | 5 | 10.2 | 49 | 100.0 |  |
| Adulthood | 41 | 68.3 | 7 | 11.7 | 6 | 10.0 | 6 | 10.0 | 60 | 100.0 |  |
| Childhood and Adulthood | - | - | - | - | - | - | 1 | 100.0 | 1 | 100.0 |  |
| **Total** | **133** | **68.9** | **21** | **10.9** | **22** | **11.4** | **17** | **8.8** | **193** | **100.0** |  |
|  |  |  |  |  |  |  |  |  |  |  |  |
| **Children** |  |  |  |  |  |  |  |  |  |  | p^(1)^ = 0.048* |
| Yes | 129 | 70.9 | 22 | 12.1 | 15 | 8.2 | 16 | 8.8 | 182 | 100.0 |  |
| No | 44 | 61.1 | 8 | 11.1 | 15 | 20.8 | 5 | 6.9 | 72 | 100.0 |  |
| **Total** | **173** | **68.1** | **30** | **11.8** | **30** | **11.8** | **21** | **8.3** | **254** | **100.0** |  |
|  |  |  |  |  |  |  |  |  |  |  |  |
| **Recent psychological treatment** |  |  |  |  |  |  |  |  |  |  | p^(1)^ = 0.846 |
| Yes | 29 | 63.0 | 6 | 13.0 | 6 | 13.0 | 5 | 10.9 | 46 | 100.0 |  |
| No | 144 | 69.2 | 24 | 11.5 | 24 | 11.5 | 16 | 7.7 | 208 | 100.0 |  |
| **Total** | **173** | **68.1** | **30** | **11.8** | **30** | **11.8** | **21** | **8.3** | **254** | **100.0** |  |
|  |  |  |  |  |  |  |  |  |  |  |  |

(*) P<0.05

(1) Pearson’s chi-squared test

(2) Fisher's exact test
